# Supplementary material for: The interactome of intact mitochondria by cross-linking mass spectrometry provides evidence for coexisting respiratory supercomplexes
Source: Mol Cell Proteomics. 2017 Dec 8;17(2):216–32. doi: 10.1074/mcp.RA117.000470 (PMC5795388; doi:10.1074/mcp.RA117.000470)
Supplement: Supplemental Data [file supp_RA117.000470_133922_0_supp_23978_fzffwf.zip › spectra_annotation/mito_DR_spectra_annotation/156-1-2-1-5-1.pdf]

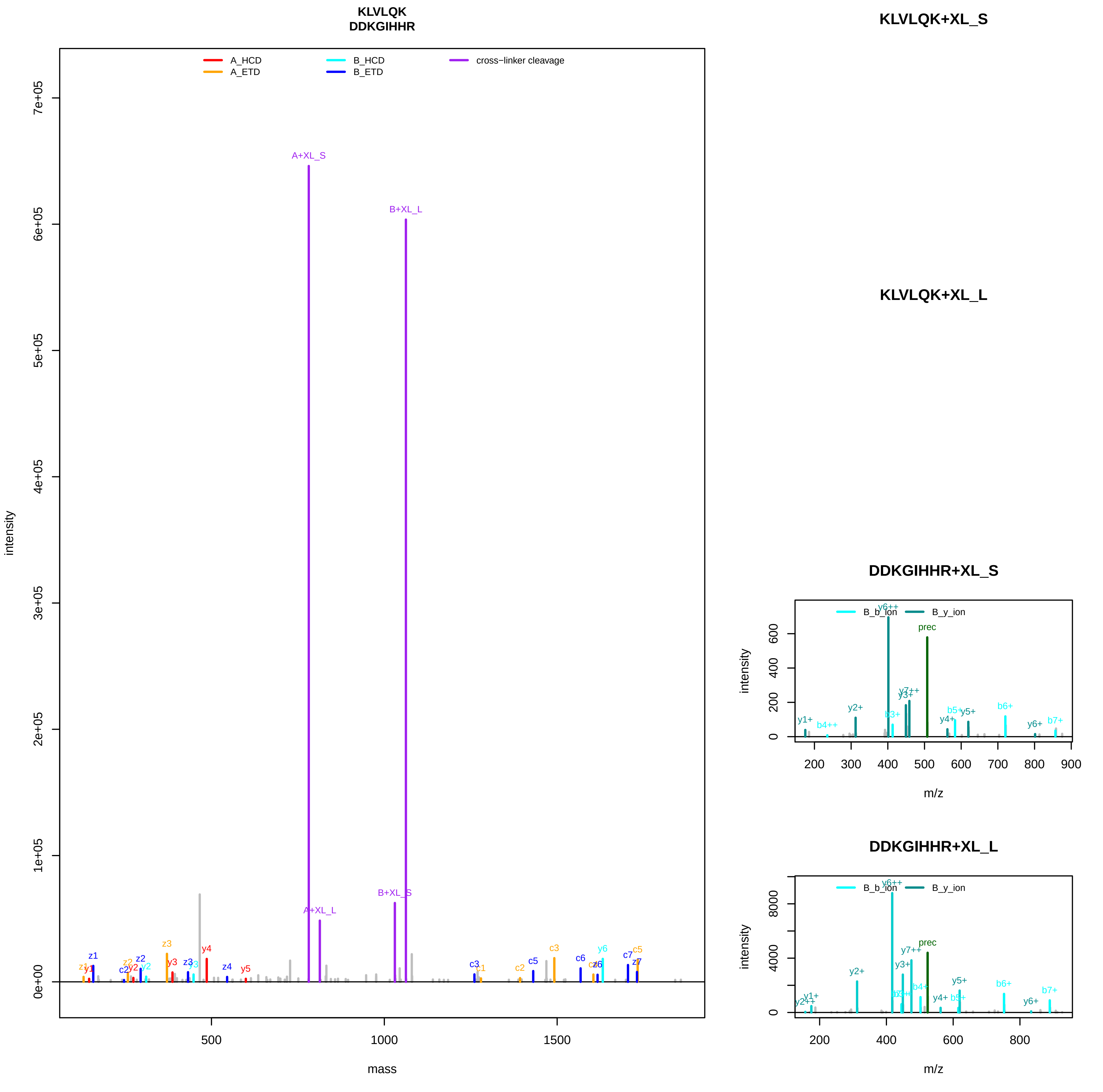
KLVLQK
DDKGIHHR
A_HCD
A_ETD
B_HCD
B_ETD
cross−linker cleavage
A+XL_S
B+XL_L
B+XL_S
A+XL_L
intensity
mass
0e+00
1e+05
2e+05
3e+05
4e+05
5e+05
6e+05
7e+05
500
1000
1500
z1
y1
c2
z2
y2
z3
y3
y4
z4
y5
c3
c2
c5
c3
c6
z6
y6
c7
z7
c5
KLVLQK+XL_S
KLVLQK+XL_L
DDKGIHHR+XL_S
B_b_ion
B_y_ion
prec
y6++
y7++
y3+
y2+
b3+
y1+
b4++
b5+
y5+
y4+
b6+
y6+
b7+
intensity
m/z
0
200
400
600
200
300
400
500
600
700
800
900
DDKGIHHR+XL_L
B_b_ion
B_y_ion
y6++
prec
y7++
y3+
y2+
y1+
y2++
b4+
y4+
b5+
y5+
b6+
y6+
b7+
intensity
m/z
0
4000
8000
200
400
600
800
